# Exploring Knowledge, Beliefs, and Attitudes of Older Adults About Prescription Opioids

**DOI:** 10.1093/geroni/igab046.872

**Published:** 2021-12-17

**Authors:** Noell Rowan, Tamatha Arms, Susan Glose

**Affiliations:** 1 University of North Carolina Wilmington, Wilmington, North Carolina, United States; 2 UNC Wilmington, Wilmington, North Carolina, United States; 3 UNC Wilmington, UNC Wilmington, North Carolina, United States

## Abstract

Over the past two decades, opioids have been considered important and acceptable in the treatment of pain for older adults, especially for chronic health conditions. Despite the fact that older adults are prescribed opioid medications at high rates, there is little research examining older adults’ knowledge, beliefs, and attitudes about opioid medications. The purpose of this study was to explore the knowledge, beliefs, and attitudes surrounding prescription opioid medications of community living older adults in a southeast area of the United States. A cross-sectional, descriptive, anonymous survey design of participants aged 55 or over was used. Study participants (N=119) reported bias in their attitudes and beliefs about the use and misuse of prescription opioid medications. Multiple regression analyses revealed that gender, age, work, marital status, and education level all had significant results in explaining variance in the statistical models. Even though study participants demonstrated high levels of education and understanding of the potential of addiction to opiates, there were a number of misconceptions revealed about prescription pain medications. This urges the necessity of increased awareness via further research, presentations, and creative discourse to assist in the understanding of precursors of addiction and ways to deal with pain that do not automatically rely on prescription opioid medicines. Implications include outreach to a larger and more diverse sample to address knowledge, beliefs, and attitudes surrounding prescription opioid medications of community living older adults.

